# The Effects of Ca^2+^ Concentration and E200K Mutation on the Aggregation Propensity of PrP^C^: A Computational Study

**DOI:** 10.1371/journal.pone.0168039

**Published:** 2016-12-13

**Authors:** Alessandro Marrone, Nazzareno Re, Loriano Storchi

**Affiliations:** 1 Università “G d’Annunzio” di Chieti-Pescara, Department of Pharmacy, Chieti, Italy; 2 Molecular Discovery Limited, Middlesex, London, United Kingdom; Russian Academy of Medical Sciences, RUSSIAN FEDERATION

## Abstract

The propensity of cellular prion protein to aggregation is reputed essential for the initiation of the amyloid cascade that ultimately lead to the accumulation of neurotoxic aggregates. In this paper, we extended and applied an already reported computational workflow [Proteins 2015; 83: 1751–1765] to elucidate in details the aggregation propensity of PrP protein systems including wild type, wild type treated at different [Ca^2+^] and E200K mutant. The application of the computational procedure to two segments of PrP^C^, i.e. 125–228 and 120–231, allowed to emphasize how the inclusion of complete C-terminus and last portion (120–126) of the neurotoxic segment 106–126 may be crucial to unveil significant and unexpected interaction properties. Indeed, the anchoring of N-terminus on H2 domain detected in the wild type resulted to be disrupted upon either E200K mutation or Ca^2+^ binding, and to unbury hydrophobic spots on the PrP^C^ surface. A peculiar dinuclear Ca^2+^ binding motif formed by the C-terminus and the S2-H2 loop was detected for [Ca^2+^] > 5 mM and showed similarities with binding motifs retraced in other protein systems, thus suggesting a possible functional meaning for its formation. Therefore, we potentiated the computational procedure by including a tool that clusterize the minima of molecular interaction fields of a proteinand delimit the regions of space with higher hydrophobic or higher hydrophilic character, hence, more likely involved in the self-assembly process. Plausible models for the self-assembly of either the E200K mutated or Ca^2+^-bound PrP^C^ were sketched and discussed. The present investigation provides for structure-based information and new prompts that may represent a starting point for future experimental or computational works on the PrP^C^ aggregation.

## Introduction

The pathogenic conversion of prion protein (PrP^C^) is one of the most representative examples of a natively folded protein that in response to detrimental stimuli may acquire aggregation propensity, form fibril deposits, and ultimately cause neurodegenerative disorders [1, 2] The misfolded or “scrapie” isoform (PrP^Sc^) has been identified as the major component of prions: the infectious entities of transmissible spongiform encephalopathy (TSE) [3, 4]. Misfolding is caused by preformed or infectant PrP^Sc^ structures that are able to recruit PrP^C^ units and catalyze their conversion to the scrapie conformation [5]. The formation of PrP aggregates and their pathogenic potential have been extensively investigated and key structural determinants of this detrimental process have been unveiled [6–10] Among the number of proposed mechanisms ([11], and ref. 4, 28, 97, 98, and 193 therein), the nucleated conformational conversion (NCC) of Lindquist seem to able to provide explanation to most of the accumulated evidences about PrP aggregation and amyloid transition [12]. According to this model, PrP^C^ aggregates to yield nuclei, i.e. poly(PrP^C^), that accumulate and slowly convert to poly(PrP^Sc^) nuclei. The “scrapie” nuclei assemble more rapidly by forming larger aggregates and act as infectious species by catalyzing the poly(PrP^C^)→poly(PrP^Sc^) conversion. The aggregation propensity of PrP^C^ turns to be as important as that of PrP^Sc^ and might be essential for the initiation of the amyloid cascade. In this view, a pathogenic stimulus of the PrP^C^→PrP^Sc^ conversion can be newly interpreted as any structural event not necessary implying a massive unfolding but rather prompting PrP^C^ into an aggregation-prone status in which secondary and tertiary structures can be mostly maintained.

Among the recognized amyloidogenic stimuli, we have recently focused our attention on two agents that mostly affect the distribution of surface charges on the C-term globule domain of PrP^C^, i.e. the well known pathogenic E200K mutation [13, 14] and the treatment of wild type PrP^C^ with moderate Ca^2+^ concentration [15–18]. By the use of a newly developed computational approach, we have eventually evidenced that both Ca^2+^ concentration and E200K mutation induce a similar electrostatic asset and a similar increased aggregation propensity in the structure of PrP^C^ [18], although several aspects are still to be elucidated: Which intermolecular forces do mainly determine the PrP^C^ self-assembly? Which residues do play a major role in the self-assembly of PrP^C^, and are the same or different residues involved in either wild type, E200K mutant, or Ca^2+^-bound PrP^C^ aggregation? May or not the pathogenic effect of [Ca^2+^] be exerted at the molar concentrations typically detected in the nervous systems?

In this paper, we used a computational approach, mostly based on the already reported computational workflow [18], to elucidate in details the aggregation propensity of PrP protein systems including wild type, wild type treated at different [Ca^2+^] or E200K mutant. Several elements of novelty characterize the present investigation compared to the previously reported one [18]: i) two PrP segments, namely 125–228 and 120–231, were used to model 90–231 PrP^C^ systems, their responsivity to the either E200K mutation of Ca^2+^ treatment were tested; ii) The effect of Ca^2+^ at different concentrations, i.e. 5, 10, and 20 mM, was monitored to model the progressive structural modifications, with the associated increase of aggregation propensity, induced by the Ca^2+^ binding at PrP^C^; iii) The computational tools for the analysis of molecular interaction properties were potentiated to identify regions of PrP surrounding space involved in the aggregation process; we showed how electrostatics and hydrophobic/hydrophilic character of the PrP^C^ surface or outer space may be used to draw hypotheses on the protein self-assembly.

This study provided for a base of structural information on which aggregation hypotheses can be sketched by the combination/harmonization with the known phenomenological constraints featuring the PrP assembly, and may be a starting point for either experimental or computational investigations on the PrP^C^ aggregation.

## Methods

### Computational workflow

The computational workflow adopted in this work to investigate the molecular interaction properties of PrP systems is partially analogous to the one already reported in our previous work [18].

**Fig 1:**
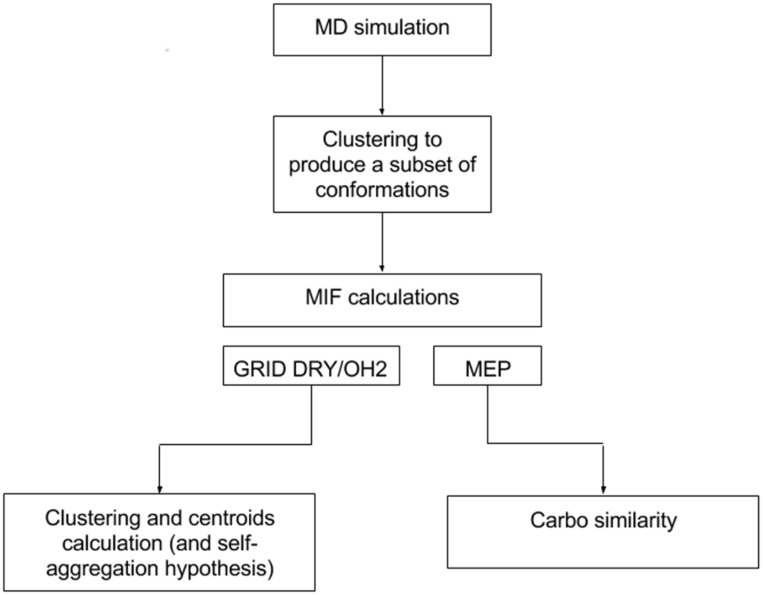
Computational workflow for the estimation of the interaction properties of a protein system.

Updates in the modelling of PrP^C^ system as well as newly developed and implemented computational procedures were adopted to increase reliability in the representation and visualization of self-interaction properties, and in the identification of the protein residues mostly or potentially involved in the PrP^C^ aggregation. Again, the very first step of the workflow is represented by a molecular dynamics (MD) simulation by which a multiconformational representation of each protein system is gained (see below). The clustering of 1000 protein conformations per trajectory ensemble is performed to select a subset of representative configurations based on the diverse distribution of the charges residues. It is worth noting that each element of a resulting subset is assigned with an intrinsic weight correlated to the size of the cluster it represents, i.e. to the number of protein conformations composing each cluster. After local minimization and alignment, the representative subsets are ready to be subjected to the analysis of molecular interaction properties that, as shown, follows two parallel branches of the workflow (Fig 1). By one side, the electrostatic properties affecting the considered PrP^C^ systems are analyzed through the calculation of the molecular electrostatic potential (MEP) and compared in terms of MEP cross-similarity using the Carbò index (CI) [18, 19]. The MEP calculation and subsequent CI computation have been performed by using an in-house utility developed in Python [18, 20]. As previously stated [18], because the HF-FMO [21] atomic charges calculated via Mulliken population analysis produced no significant deviations with respect to the OPLS atomic charges in the present work, we used only the latter to compute the Carbò index, as reported in the workflow of Fig 1. By the other side, the hydrophobic/hydrophilic character of the PrP surface is estimated by using a GRID force fields [22, 23] calculation. We used the GRID MIF to sketch self-aggregation hypotheses based on the identification of the minima of either the DRY and HOH probes. Subsequently, these points are clusterized by a K-means algorithm [24, 25] to locate regions of the protein surrounding space with an either hydrophobic or hydrophilic character. We may adopt also a different approach to determine the optimal rotation matrix that minimize the RMSD between two sets of centroids that is the Kabash algorithm [26]. The visualization of these regions allows to easily sketch hypotheses of self-aggregation by the use of a graphical-user interface. Details on the selected PrP^C^ systems, MD simulation, subset generation, and MIF calculation and analysis are provided in the supporting information (S2 File).

## Results

**Fig 2:**
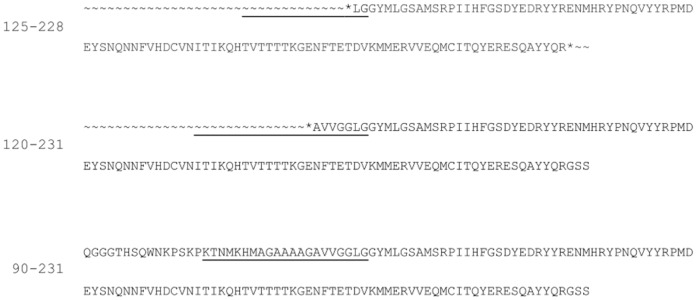
Comparison of PrP primary structures. Segment a (125–228,top), segment b (120–231, middle), and whole 90–231 segment(down) of the human prion protein. * indicates amide capping by acetyl or N-methyl amide of N or C terminal, respectively. The neurotoxic peptide segment 106–126 is underlined.

The present computational investigation of 90–231 PrP^C^ protein systems was carried out by employing experimentally determined 3D structures of 125–228 (Segment a from now on) and 120–231 (Segment b from now on) segments (see Fig 2 for details) as starting models. Despite segment a and b are rather similar and differ by only a few amminoacids, they could display diversities in the aggregation properties and in the response to either mutation or [Ca^2+^] treatment.

More specifically, the effects of increasing [Ca^2+^] concentration and E200K mutation were investigated on the PrP systems summarized and labeled in Table 1 by adopting a computational scheme analogous to the one reported in ref. [18].

**Table 1:** Summary of the investigated PrP systems and their labels.

Stimuli	PrP systems	
	Segment a	Segment b
wt	Ia	Ib
E200K	IIa	IIb
wt, [Ca^2+^] = 5 mM	IIIa	IIIb
wt, [Ca^2+^] = 10 mM	Iva	IVb
wt, [Ca^2+^] = 20 mM	Va	Vb

### MD analyses

Molecular dynamics simulations were performed to gain 100 ns of equilibrated trajectory for each system. The analysis of the positional displacement of backbone and ionizable residues showed an adequate equilibration within the range 100-200ns (S1 Fig). All systems maintained in the cellular form in the considered time window.

Notable peculiarities in the structural asset of segment b were detected in the C-terminal region of H2 (PrP^C^ domains are labelled as in ref. [18]). We noticed that only in system Ib the 120–125 frame (N-terminus) is anchored to the H2 segment 184–189 through hydrophobic contacts while either E200K mutation (IIb) or Ca^2+^ binding (IIIb-Vb) seem able to alter or disrupt this anchoring. The N-terminus in the E200K mutant was anchored on H2 by polar contacts thus causing the exposure of hydrophobic residues to the solvent; in Ca^2+^bound systems such anchoring was disrupted and the N-terminus was rather involved in interactions with S2 (Tyr 128, Leu 130) or H2-S2 (Arg 164, Pro 165) or H2 (Gln 186) mainly via hydrogen bonds (S2 Fig). We also detected that the threonine-rich motif TTTTK on H2 (190–194 segment) is more elongated in Ib when N-terminus anchoring occurs, whereas this segment is less elongated in all other systems.

**Fig 3:**
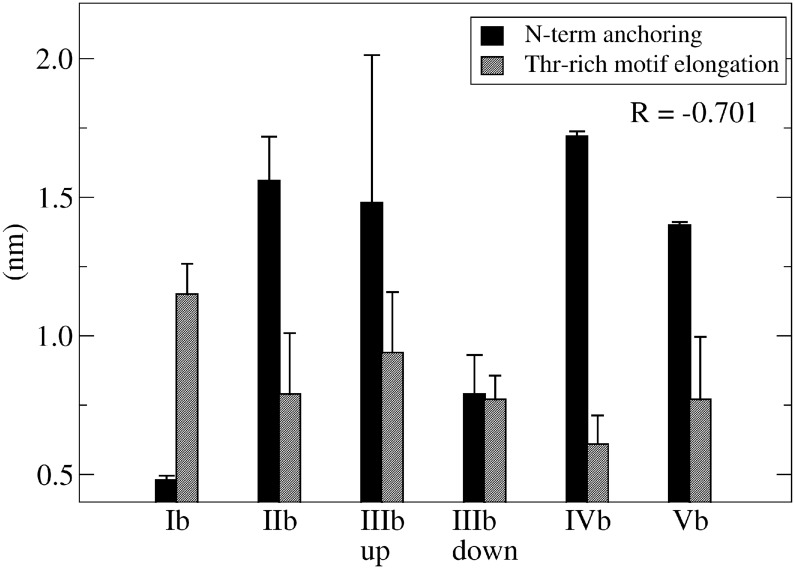
Correlation between N-terminus anchoring and threonin-rich motif elongation at H2 domain. Anchoring of the N-terminus expressed as the average distance between residues 122–123 and 184–189 and elongation of threonin-rich motif expressed as the average distance between residues 190 and 194. Standard deviation bars and the regression coefficient (R) between the two data sets were also reported.

To shed some light on the possible correlation between the anchoring of N-terminus and the conformation of the H2 threonin-rich motif, we compared the average distance between the center of mass of residues 122–123 and 184–189 with the average distance between residues 190 and 194. As shown (Fig 3), the anchoring of N-terminus and the elongation of threonin-rich motif were found to be slightly (negatively) correlated thus suggesting that these structural features could be subjected to a reciprocal modulation.

**Fig 4:**
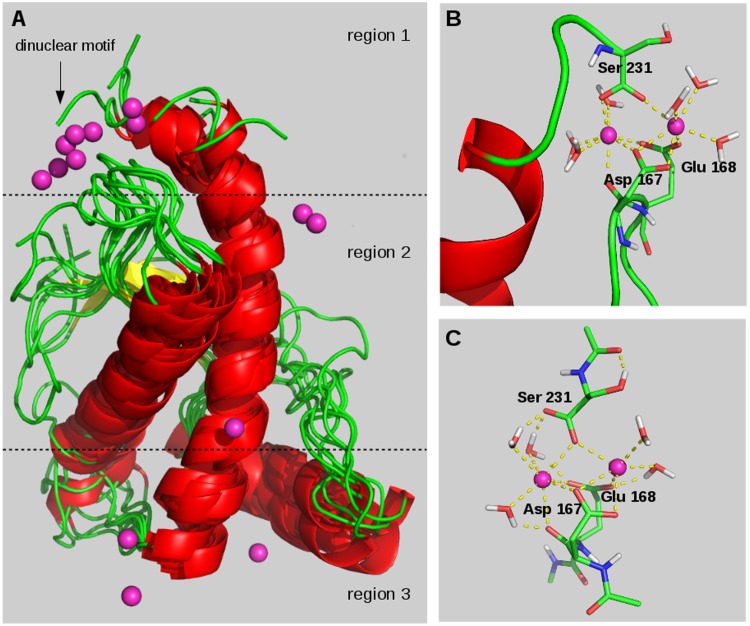
The Ca^2+^-coordinating sites on the PrP structure. (A) Distribution of Ca^2+^ coordination sites by the superposition of all III-V systems. Details of the dinuclear coordination motif detected in region 1 obtained by (B) molecular dynamics simulation and (C) quantum mechanical optimization of a reduced model retrieved from system Vb.

The calculation of the minimum protein-Ca^2+^ distances was then performed to identify the groups of Ca^2+^-coordinating residues in all the III-V systems. The PrP structure and its surrounding space were then partitioned in three region, according to the distribution of Ca^2+^ binding sites along the major axis of the protein fold (Fig 4).

#### Region 1

Ca^2+^ coordination site proximal to the S2-H2 loop and formed by Asp 167 and Glu 168. Interestingly, only in IVb and Vb this coordination site gave rise to a peculiar dinuclear Ca^2+^ binding motif (DBM) (Fig 4B) because it could be formed when more than one Ca^2+^ ion is present in the system and the carboxylate terminus of PrP is complete. Indeed, DBM is formed by the Asp 167 and Glu 168 side chains, by the carboxyl terminus of Ser 231, and by 5–6 water molecules completing the hexacoordination of each Ca^2+^ ion. To further corroborate the formation of this coordination motif, quantum mechanical (QM) calculations (S2 File) were performed on a reduced model of DBM formed by residue 167, 168, and 231, and by five of the coordinated waters. The QM optimized structure of DBM model resulted to be largely similar to the one extrapolated from trajectory with only a slight rearrangement of Ser 231 carboxylate terminus orientation (Fig 4C).

#### Region 2

Ca^2+^ binding residues were only found on the C-terminal segment of H3 (207–221).

#### Region 3

Ca^2+^ coordination were mostly placed in the region spanning over the H2-H3 loop (Asp 196, Glu 200, Glu 202) and H1 (Asp 152).

From the fact that in the presence of one Ca^2+^ ion in the simulation box (systems III) only region 1 and 3 resulted to be coordinated, we gained an indirect evidence of the higher Ca^2+^-affinity of these regions compared to region 2. The analysis of the occurrence of Ca^2+^-binding events along the trajectories of systems III-V confirmed this hypothesis by showing that region 1 binds at least one Ca^2+^ ion for a high (> 70%) extent of trajectory and in all systems. When only one Ca^2+^ ion in present in the simulation box, region 1 and region 3 were coordinated, while coordination at region 2 was detected only at higher [Ca^2+^] (S1 Table).

**Fig 5:**
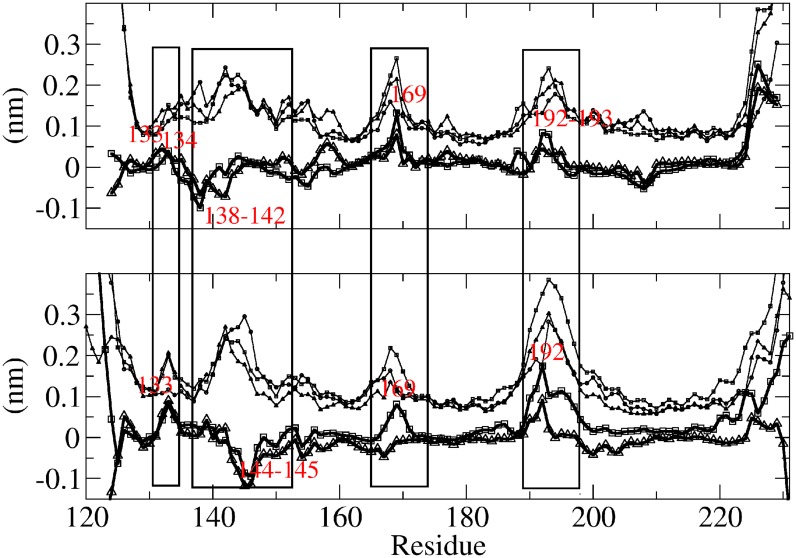
Atomic fluctuations of PrP backbone. Root mean squared fluctuations per residue (rmsf) for I (circle), II (square), and V (triangle) systems and II-I (bold, square) and V-I (bold, triangle) rmsf difference per residue (Δrmsf); a and b systems reported on top and bottom, respectively. Boxes highlight the residues with Δrmsf values outside the range of −0.05–0.05 nm (dashed lines).

The effects of either mutation or Ca^2+^ treatment on the atomic fluctuations of PrP backbone were also reported for I, II, and V systems. The profiles of root mean squared fluctuation (rmsf) per residue were substantially similar to those previously reported (compare with Fig 5 in ref. [18]), showing for all systems an expectedly higher fluctuation of terminal residues and lower values for S1, S2, H2, and H3 domains compared to H1, S2-H2 loop, and H2-H3 loop (Fig 5).

By the analysis of Δrmsf profiles we were able to highlight increase or decrease of residue fluctuations in response to mutation or Ca^2+^ coordination [18], mainly unveiling significant changes in the fluctuation of four groups of residues (Fig 5). Both stimuli slightly decreased the fluctuation of H1 residues, e.g. 138–142 and 144–145 for segment a and b, respectively, while increased the fluctuation of residues around 133–134, 169, and 192–193, as already ascertained previously [18]. The lack of and the lower fluctuation increase around residue Tyr 169 and Thr 192 of system Va and Vb, respectively, were the only detected differences between 125–228 and 120–231 systems.

Sets of 1000 (1:100 ps) snapshots taken from the last 100 ns of each trajectory were clusterized by the root mean squared deviations of the atomic coordinates (rmsd) within the subgroup of ionizable aminoacids Glu, Asp, Arg, Lys, and His. This clustering analysis provided for a subset of representative conformations disclosing the various configurations of surface charges in a thermally equilibrated PrP molecule; the elements of the representative subset correspond to the middle structures of each cluster (S2 File). A summary of clustering analyses is also available (S2 Table).

### MEP analyses

**Fig 6:**
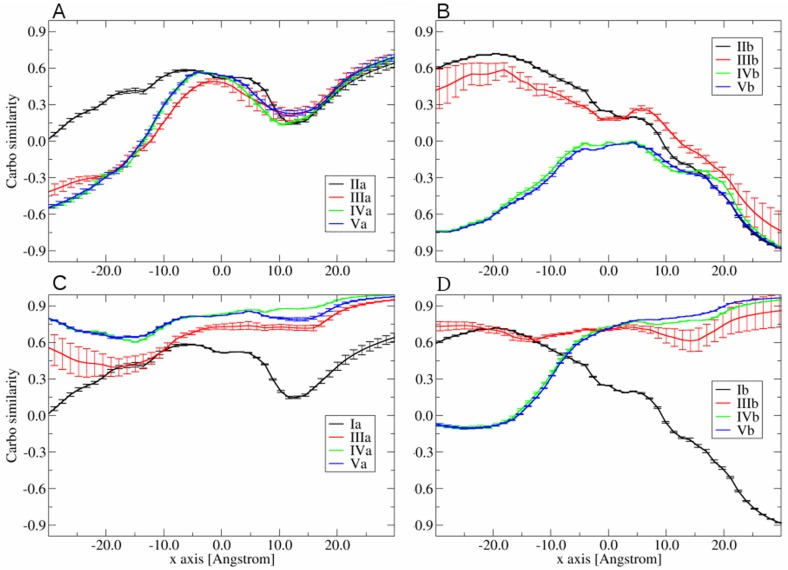
MEP similarity. Profiles of Carbò index compared each PrP system versus I (A-B) and II (C-D) systems. The reported IIIa and IIIb profiles were obtained by averaging IIIa1\IIIa2 and IIIb1\IIIb2 profiles, respectively.

The molecular electrostatic potential (MEP) was employed to retrace the electrostatic properties of the investigated PrP systems. MEP is probed in the PrP surrounding space of each subset elements by using a grid-based approach after assigning the corresponding OPLS charge to the protein atoms and, in the case of Ca^2+^-bound systems, to the atoms of the Calcium-coordinated water molecules. The pairwise comparison of MEP within the investigated PrP subsets was performed through the calculation of the Carbò similarity index (CI) as previously reported [18]. In such a scheme, CI was calculated for all the grid points at the same x coordinate, i.e. laying on yz planes, thus providing for a CI profile that scans the electrostatic similarity between two proteins along the x axis. The similarity profiles obtained by the comparison of Ia with all the other systems IIa-VIa were substantially in agreement with those previously reported [18], spotlighting three regions of major impact in these structural comparisons: i) the space surrounding the C-terminus and S2-H2 loop, x < −10; ii) the space stacked on and off the laying plane of H2, x = 0; iii) the space proximal to the mutation site, x > 10, including the H2-H3 loop and part of the H1 domain (Fig 6A).

These regions of MEP profiles are consistent with the three regions of PrP identified by the analysis of the Ca^2+^ binding sites on PrP. In region 1, the trend of MEP similarity is decreasing as a result of the overall charge inversion induced by either E200K mutation or Ca^2+^binding. In the middle segment of CI profile (region 2) a certain degree of similarity is maintained in a narrow range of CI (0.3–0.5) for all comparisons, thus indicating that the MEP in this region is not appreciably affected by mutation or Ca^2+^ binding. Local minima of CI were instead detected around x = 10–15 (region 3) in the profiles comparing Ia with all other systems, in agreement with previous results [18]. Interestingly, the local minima of CI in comparison with Ia were in the trend IIa~IVa<IIIa~Va, suggesting that the changes of MEP in region 3 may accumulate by increasing [Ca^2+^] but are saturated for [Ca^2+^] > 10 mM. The profiles of MEP similarity to Ib were also divided in three region of interests, resembling the x value ranges reported for Ia-VIa although with diverse shapes and ranges (Fig 6B). The region 1 of IIb-Ib and IIIb-Ib profiles corresponds to the x < −10 segment and ranges at positive CI values (0.3–0.6). On the other hand, the same segment for VIb-Ib and Vb-Ib comparisons ranges at negative CI values (−0.3-−0.6), probably because of the neat charge inversion therein induced by the dinuclear Ca^2+^ coordination for [Ca^2+^] > 5 mM (systems IVb and Vb). Also in these profiles, the middle segment around x = 0 (region 2) was characterized by an almost horizontal trend, hence the IVb-Ib and Vb-Ib profiles, by one side, and IIb-Ib and IIIb-Ib profiles, by the other, ranged around 0.0 and 0.15 CI units, respectively (Fig 6B). At variance from segment a profiles, monotonic decreasing trends were detected in place of local minima for all segment b comparisons in region 3. The effects of [Ca^2+^] on MEP similarity profiles accumulate and saturate by almost overlapping on the IIb-Ib profile for [Ca^2+^] ≥ 10 mM (Fig 6B). This result was further corroborated by the CI profiles obtained by the comparison of all systems to IIa or IIb that displayed a progressive increase of similarity by increasing [Ca^2+^] but saturated for [Ca^2+^] ≥ 10 mM (Fig 6C and 6D). Despite the overall comparison of MEP similarity outcomes indicated that segments a and b disclose a quite similar response to E200K mutation or [Ca^2+^], i.e. in terms of increase/decrease similarity, some notable differences were detected in the shape of CI profiles that seems to be more strictly dependent on the PrP segment. In particular, the opposite trends of MEP similarity found for x > 15 (far region 3) probably reflect a diverse structural responsiveness of segments a and b to either mutation or [Ca^2+^] and stress that the extension by even a few residues allowed to appreciate a different responsivity in the PrP models.

**Fig 7:**
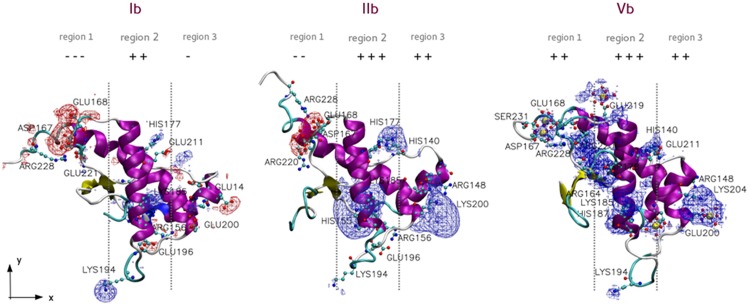
Averaged MEP isosurfaces. Reported PrP were Ib (left), IIb (middle), and Vb (right) subsets. Positive and negative isosurfaces are depicted as blue and red meshes, respectively. Qualitative insight of the charge amount per region is also reported.

To better describe how the investigated stimuli affect the PrP electrostatics, theMEP of I, II, and V molecular systemsfrom either a and b segment were calculated by averaging over the MEP of each subset element thus obtaining a3D map of this property (S2 File). Assigned negative or positive values of average MEP can be then visualized as isosurfaces in the PrP surrounding space. The visualization of MEP isosurfaces have been already used to differentiate the electrostatics of wild type and E200K mutated PrP^C^ but applied on single PrP conformation [14]. Here, the isosurface representation was in fact applied to an average MEPthatmediatesthe effects of thermal and solvent-induced dynamics on PrP electrostatics. In particular, we aimed at spotlighting the protein frames with higher positive or negative charge density and appreciating the most significant changes in the charge patterns on PrP in response to either E200K mutation or Ca^2+^ treatment. Despitesome differences in the distribution and extension of MEP isosurfaces ofsegment a and b (for more details see S3 Fig and S1 File), the overall effects of either E200K mutation or Ca^2+^ treatment can be appreciated by focusing on the MEP isosurfaces calculated on Ib, IIb, and Vb systems (Fig 7).

As expected, the local charge inversion induced by E200K mutation led to a massive increase of the positive charge mainly in region 3 (marginally in region 2), and toonly a slight decrease of negative charge in region 1. Thus, E200K mutation inverts the electrostatic character of region 3 and, consequently, confers electrostatic complementarity to region 1 (negative) and 3 (positive) (Fig 7 middle). The binding of Ca^2+^ ions induced a generalized increase of the positive character of the PrP and only positive isosurfaces were detected. However, large positive isosurfaces were still detected around protein side chains rather than surrounding the Ca^2+^ coordination sites (Fig 7 right). Indeed, to our analysis, both region 1 and region 3 coordination sites of Vb were substantially neutral, presenting only small positive and tightly limited isosurfaces aroundthe Ca^2+^ centers. Thus, the Ca^2+^ coordination sites on Vb are not appreciably charged and their spatial approach would be not or only slightly disfavored by electrostatics.

### Ca^2+^-mediated aggregation

**Fig 8:**
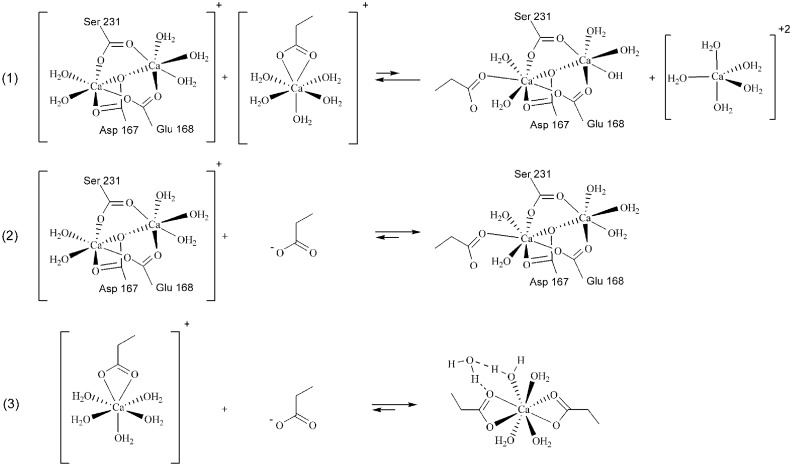
Ca^2+^-mediatedaggregation of PrP^C^ units. Hypotheses drawn for the reaction of DBM with free or [Ca(H_2_O)_5_]^2+^-coordinated CH_3_CH_2_COO^-^ (reaction (1) and (2), respectively), and reaction of [Ca(H_2_O)_5_(CH_3_CH_2_COO)]^+^ with CH_3_CH_2_COO^-^ (3). Reduced model of DBM was obtained by replacing Ser 231, Asp 167, and Glu 168 labels with the corresponding aminoacid scaffold in the capped form: N-acetyl and methylamide form of N and C terminus, respectively. Monocoordinated Ca^2+^-binding sites were modelled by CH_3_CH_2_COO^-^.

As shown, while E200K mutation increases the self-interaction of PrP^C^ essentially by inducing electrostatic complementarity of region 1 and 3 (more evident on IIb), the same effect cannot be ascribed to [Ca^2+^]. On the other hand, we noticed that region 1 and 3 together host the majority of Ca^2+^ coordination sites and, notably, in close proximity to the complementary interfaces detected on E200K mutants. Also, our analyses showed that a minor extent of positive isosurfacesis located on the Ca^2+^ coordination sites. On these bases, we hypothesized that Ca^2+^ coordination itself may be the structural frame responsible of the PrP^C^ self-aggregation: the approach of two coordination sites may lead to a ligand rearrangement, and, eventually, to the formation of an interunit Ca^2+^ complex. Quantum chemical calculations were performed to investigate the thermodynamics of the possible Ca^2+^-mediatedcondensation of reduced models of region 1 and 3 extracted from the Vb system (Fig 8).

The reaction of DBM with propanoate coordinating one [Ca(H_2_O)_5_]^2+^ moiety resulted to be endoergonic by 1.5 kcal/mol, thus corresponding to a slightly left-shifted equilibrium (Fig 8, (1)). This process would resemble the approach of DBM with a Ca^2+^-bound carboxylate, i.e. any other Ca^2+^ coordinating group on PrP^C^, inducing ligand exchange and release of a [Ca(H_2_O)_5_]^2+^ moiety in the bulk. On the other hand, the coordination of a free propanoate groupat DBM, i.e. a model of not coordinated Glu or Asp side chains coordinating at the dinuclear motif, was found to be exoergonic by -26 kcal/mol, thus indicating that process (2) is largely favored. Also, the formation of a dicarboxylate complex by the further coordination of a carboxylate ligand (reaction (3), Fig 8) is exoergonic by -21.5 kcal/mol; this process may resemble the interunit approach between an uncoordinated Asp or Glu residue with an occupied Ca^2+^-binding sites and, thus, represent a further example of Ca^2+^-mediated interaction occurring in the PrP self-assembly.

### MIF analyses

**Fig 9:**
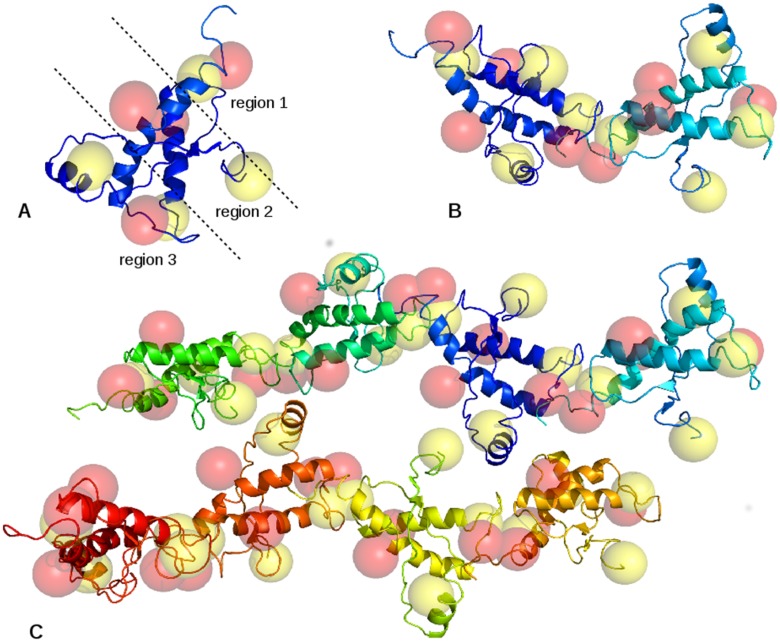
Hypothesized structures of E200K-PrP^C^ aggregates. (A) Representation of the E200K-PrP^C^ with its clusters of DRY (yellow) and HOH (red) minima. (B) Two E200K mutants chained through region 1-region 3 interactions. (C) Interaction of two E200K tetramers by the approach of hydrophobic clusters on region 2.

The interaction properties of Ib, IIb, and IVb systems were assayed by the calculation of grid-based molecular interaction fields (MIFs) by the use of two almost complementary probes, i.e. DRY and HOH (S2 File) [22], providing for an estimation of the hydrophobic and hydrophilic interaction, respectively, in the PrP surrounding space. This newly developed procedure of MIF mapping was designed to locate regions with a high hydrophobic or hydrophiliccharacter in the surrounding space ofa protein molecule. Either DRY or HOH mapswere calculated for each structure of the protein subset and the corresponding local minimum points were identified. These points were then grouped in terms of their spatial proximity, thusforming clusters of DRY or HOH minima which can be straightforwardly visualized and inspected. The outcomes of this procedure applied to system IIb are reported (Fig 9).

Thehydrophobicorhydrophilicclustersare represented by yellow or red spheres, respectively, that delimit a portion of the E200K mutant surrounding space with a high affinity for, in the same order, hydrophobic orhydrophilic counterparts. Both region 1 and region 3 resulted to be characterized by one hydrophilic and one hydrophobic cluster similarly far apart, i.e. 7.1 and 8.2 Angstrom, respectively, and well exposed to the bulk (Fig 9A). The cluster of DRY minima in region 1 mainly resulted from proximal tyrosine residues, i.e. Tyr 218, Tyr 225, and Tyr 226, and from Met 166. On the other hand, the hydrophilic cluster in region 1 was mainly ascribed toAsp 167, Glu 168, and Glu 221, also identified as Ca^2+^ binding residues, and Gln 227. Region 2 presented two hydrophobic and two hydrophilic clusters. The latter, one at S1-H1 loop and one close to Glu 219 on H3, are both near the region 1-region 2 boundary. The hydrophobic cluster proximal to the N-terminus is mostly ascribed to Val 121 and Val 122, whereas the one at the interface between H1 and S1-H1 loop includes Phe 141, Tyr 150, and Met 205. The two complementary clusters in region 3 are proximal to the H2-H3 loop (hydrophilic) and the threonin-rich motif of H2 (hydrophobic). The former includes mainly Glu 196 and Arg 156 involved in a strong ionic bridge interaction. The aromatic ring of Phe 198 was also found at the interface between this cluster and the hydrophobic one. Val 189 is the central residue of the DRY cluster in region 3 with a minor contribution of Thr 190 and Thr 191. Notably, this DRY cluster and the one placed on N-terminus (region 2) originate by the anchoring disruption (See above) causing an increase of the bulk exposure in the two detached frames (S2 Fig). By the use of a graphical interface (See ref 4 in S2 File), we rotated and translated two units of E200K and identified a zig-zag alignment in which region 1 and region 3 clusters resulted to beeffectively overlapped (Fig 9B). In this spatial configuration, the region 1-region 3 approach of E200K mutants is favored by both electrostatic interactions that are exerted at long distances and probably drive the reciprocal orientation of these PrP units, and by hydrophobic/hydrophilic matches that stabilize the self-aggregation at lower distances. Then, we assembled these E200K dimers along two approximately perpendicular directions: the one growing by region 1-region 3 overlapping, and the one growing via the lateral overlapping of the two hydrophobic clusters on region 2 (Fig 9C). As showed, this picture of E200K aggregation takes advantage of the mutual overlapping of all hydrophobic clusters which is probably the main thermodynamic drag, and of the formation of pores that host the two (unpaired) hydrophilic clusters of region 2.

An analogous picture of self-aggregation was then probed for wild type and Ca^2+^-bound PrP^C^ by the calculation of MIF clusters on Ib and IVb representative subsets, respectively. At this purpose, we aligned the protein structures of either Ib or IVb systems on the PrP units of E200K octamers to obtain analogous wild type and Ca^2+^-bound octamers, and to assay whether an analogous overlapping of MIF clusters could be appreciated. In fact, both wild type and Ca^2+^-bound PrP were characterized by distributions of MIF clusters not suitable to induce the same interunit matches detected in the E200K octamer. In the wild type octamer, only two MIF matches were detected: The one along the region 1-region 3 direction (S4 Fig, purple circles) is an effective overlapping of hydrophobic clusters although it is not reinforced by electrostatics, bearing these regions approximately the same negative character. Another match was detected between the hydrophobic cluster corresponding to the N-terminus-H2 interaction and an hydrophobic cluster placed in the middle of H1 domain (S4 Fig, dashed arrows). In the latter match, both hydrophobic clusters are poorly exposed to the bulk so that their approach is sterically disfavored. On the other hand, the alignment of Ca^2+^-bound PrP units obtained by IVb system showed that the region 1-region 3 may match by the mediation of Ca^2+^ coordination. In particular, we showed that the Ca^2+^ center of region 3 may superimpose on one Ca^2+^ ion in the DBM of region 1 (S5 Fig); the region 1-region 3 interaction would be thus yielded by a coordination exchange process resembling the reaction (1) depicted in Scheme 2. It is also worth noting the parallel approach of the Ca^2+^-coordinated Glu 219 on one unit to the carboxyl side chain of Glu 200 on the other unit that could potentially coordinate at this metal center and lead to a further stabilization of region 1-region 3 interaction. The octamer of Ca^2+^-bound PrP^C^ showed no other effective matches but rather a close proximity of MIF clusters in two regions: i) the interunit approach of the hydrophobic cluster on region 1, and ii) the approach of the hydrophobic cluster at H1 domain with that on the H2 domain (S6 Fig).

## Discussion

**Fig 10:**
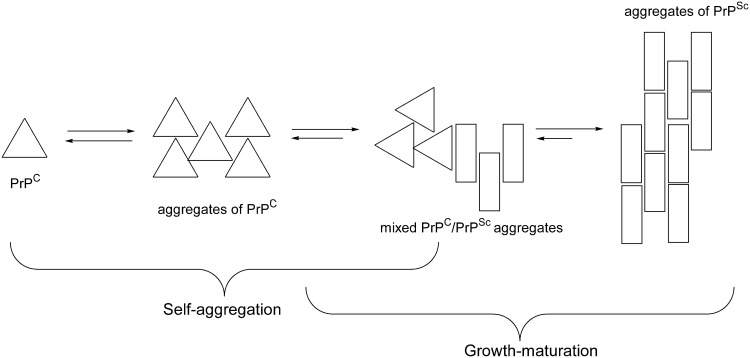
Simplified scheme of the formation of PrP aggregates.

It is generally accepted that PrP aggregation plays a pivotal role in the pathogenesis and infectivity of prions, and that the conformational rearrangement of native protein, PrP^C^, into its misfolded form, PrP^Sc^, is responsible of the high stability and inertness of the fibrillar depots [1–11]. In the line of the Lindquist model [12], two essential phases can be retraced from the multistep PrP aggregation process: i) self-interaction, ii) growth/maturation. Transient (“immature”) aggregates, corresponding to poly(PrP^C^) nuclei, are priory formed through the self-interaction of PrP^C^ units. The spontaneous formation of these “cellular” nuclei is a relatively rare event but it could be promoted by pathogenic stimuli of diverse nature, such as chemical or thermal mild denaturation, mutation, etc. In this early stage the PrP^C^ structure is substantially unchanged and an equilibrium between nuclei and single units is established. Subsequent steps of maturation yield more and more stable (“mature”) and inert aggregates of poly(PrP^Sc^) or mixed poly(PrP^C^, PrP^Sc^) nuclei with an increasingly higher PrP^Sc^/PrP^C^ ratio, while the concomitant growth of these particles may occur by the further incorporation of either monomer or multimer PrP (Fig 10).

These two stages of the PrP aggregation mechanism are sequential but overlapped in time, thus yielding to a complex variety of species in the aggregation bulk. Electrostatics and hydrophobic effect play different roles in this aggregation scheme. Electrostatics is pivotal in the early stages of aggregation by inducing the correct orientation of monomers, mainly due to its long-ranged nature, and by favoring their conformational rearrangement. The mutual approach of PrP^C^ units in the self-aggregation stage is prompted by electrostatics in which have to be included either the long-ranged attraction/repulsion between opposite/same charges or the short-ranged, highly directional H-bond or salt bridges interactions. In these early “electrostatic” aggregates each monomer structure maintains its “cellular” topology, although their mutual interactions may generate a system of electrostatic forces promoting the conformational rearrangements that gradually lead to the growth and maturation of the protein aggregates. Hydrophobic effect can be manifested only at short distances although it provides for the gross of the energy gain associated to aggregation. Self-aggregation is characterized by a small hydrophobic effect because PrP units maintain in their folded conformation, i.e. with a minimal or lesser extent of hydrophobic exposure. On the other hand, the growth/maturation step is mostly prompted by an increasing extent of hydrophobic interactions exerted by more and more unfolded PrP units.

In this line, the identification of the structural factors responsible of the prior self-interaction of PrP^C^ units may represent the first step in the development of pharmacological approaches to prevent the early stages of PrP aggregation cascade, and/or inhibit the incorporation of PrP^C^ into pre-formed aggregates, i.e. interfering with the molecular mechanisms of prion infectivity. Although a body of evidences has been collected about formation mechanisms and structural characterizations of PrP aggregates [27–32], a detailed and structure-based elucidation of how a pro-scrapie stimulus may trigger the self-interaction in cellular PrP is still missing. We have previously shown that pathogenic stimuli may induce a similar distribution of charges on the PrP surface, and that such electrostatic modifications of the protein electrostatics may lead the protein into an aggregation-prone status [17, 18].

In this paper, the structural bases driving the self-interaction of cellular PrP (90–231 PrP^C^) in response to two pathogenic stimuli, E200K mutation and Ca^2+^ treatment, were specifically investigated by the use of a newly developed computational approach combining molecular dynamics (MD) and molecular interaction field (MIF) analyses [18]. Two reduced segments were selected to model 90–231 PrP^C^ protein systems, i.e. 125–228 and 120–231 segments, named segment a and b, respectively, being their corresponding 3D structures experimentally available. The effects of [Ca^2+^] on the wild type structures of segment a and segment b were estimated at 5, 10, and 20 mM, a range of concentration already explored in experimental assays [17]. Above all, ten molecular systems, e.g. Ia-Va and Ib-Vb (Table 1), were simulated in MD calculations to yield 2 microseconds of productive trajectories. The overall secondary and tertiary structure assets (S1 Fig), placement of the Ca^2+^ binding sites (Fig 4), or per residue fluctuation patterns (Fig 5) are similarly modelled by both segment a and b systems. All systems maintained their cellular form conformation along the considered simulation time, indicating that unfolding is not prompted in short time by mutation or Ca^2+^ treatment, but it more gradually evolves after the initiation of PrP self-aggregation. Segment a and b systems treated with [CaCl_2_] (III-V) shared a similar spatial distribution of Ca^2+^ coordination sites which allowed to partition the PrP surrounding space into three regions (Fig 4): region 1 and 3 host early coordination sites, hence occupied at even low [Ca^2+^] (III systems), whereas coordination of region 2 sites occurs at higher [Ca^2+^] (V systems).

On the other hand, two significant peculiarities distinguished the segment b models of PrP in the response to either E200K mutation or Ca^2+^ treatment. Firstly only in Ib system, the 122–123 residues, C-ending the so called “neurotoxic peptide”, formed a hydrophobic cluster with 184–189 (H2) that essentially anchored the unfolded N-terminus at the globule domain. On the other hand, this anchoring is partially (IIb, IIIb) or completely (IVb, and Vb) disrupted when either one of the considered pathogenic stimuli occurred. Notably, we detected the beta-sheet pairing with S1 of the detached N-terminus in IVb and Vb, a structural modification that resembles in part the conformational asset of PrP^C^ in already reported models of PrP oligomers [33]. The N-terminus anchoring at 184–189 residues was also found to be correlated with the elongation of the vicinal threonin-rich motif 190–194, elsewhere identified as a “spot” of structural ambivalence and may be involved in the beta-sheet enrichment characterizing the cellular→scrapie conversion [34, 35]. We reputed these structural modifications able to affect the molecular interaction properties of PrP^C^. Indeed, the alteration or disruption of the N-terminus anchoring unavoidably caused a higher exposure of hydrophobic side chains, that might enhance the self-aggregation propensity of PrP. The second significant peculiarity is that a distinctive dinuclear binding motif (DBM) was detected only by the MD simulation of IVb and Vb systems, hence for [Ca^2+^] > 5 mM. The DBM can be detected only in segment b systems, because involving the carboxylate terminus of Ser 231, and, obviously, only at [Ca^2+^] > 5 mM where the Ca^2+^:PrP ratio is higher than 2:1. Parallely, it is worth taking into consideration that a maximum response in either cytotoxicity and/or conductance assays have been analogously gained for [Ca^2+^] > 5 mM [17], thus suggesting that the formation of DBM itself may have some functional meaning. Indeed, the DBM detected in region 1 of PrP^C^ showed appreciable similarities with multinuclear Ca^2+^ binding sites found in other proteins such as thermolysin [36, 37], synaptotagmin [38], and perforin [39]. Therefore, further corroboration to the DBM formation was provided by quantum mechanical calculations that substantially confirmed the coordination geometry obtained by MD calculations. The clustering of MD trajectory evidenced only slight differences affecting segment a and b systems in the response to either E200K mutation and Ca^2+^ treatment, consisting in a generally lower number of representative elements in the segment b subsets which probably reflects an extra stabilization of the protein model gained by the extension of both C- and N-terminus.

PrP systems electrostatics was then investigated through the calculation and comparison of the molecular electrostatic potential (MEP) of the representative subsets. The analysis of Carbò similarity profiles gave further consistency to the three-region partition of PrP space based on the Ca^2+^ binding site positions. All profiles showed in region 1 (x < -10) and region 3 (x > 10) the highest positive or negative slopes of Carbò similarity, thus, highlighting the presence of protein frames in these regions majorly sensitive to either mutation- or Ca^2+^-induced electrostatic changes. Notably, peptide fragments already recognized to play a role in the pathogenic conversion of PrP were detected in these two regions, namely the S2-H2 loop (region 1), and H2-H3 loop (region 3). The former is a rigid loop, prevalently in a 3_10_-helix structure conformation [40], that together with the C-terminus of the H3 domain has been reputed to form an epitope targeted by an unknown promoter of the PrP^C^→PrP^Sc^ conversion [41]. The H2-H3 loop is very close to the E200K position and, as we reported elsewhere [18], it shows an appreciably sequence similarity with the EF-hand motif of parvalbumin [42, 43]. On the other hand, low slopes were detected in all Carbò similarity profiles in the -10 < x < 10 domain corresponding to region 2; electrostatic changes induced by E200K mutation of [Ca^2+^] are seemingly less reflected in this region, or, more likely, such modifications involve uncharged residues, hence not detectable by the analysis of MEP. Despite some difference in the shapes of Carbò similarity profiles was unveiled (See Results), a consensus in the MEP analysis of segment a and b systems was the indication of region 1 and 3 as mostly affected by modifications of PrP electrostatics. The calculation of averaged MEP (Fig 7) highlighted how the poor or negligible electrostatic complementarity between region 1 (negative) and 3 (almost neutral) detected in wild type systems is, on the contrary, markedly enhanced by E200K mutation whose major effect was the increase of the positive charge on region 3 and, marginally, region 2. The E200K mutants are thus characterized by an intrinsic electrostatic complementarity that would favor the PrP^C^ self-interaction through the approach of region 1 and 3. As expected, the progressive occupation of Ca^2+^ binding sites led instead to a global increase of positive charges on the PrP surface that in fact would inhibit or disfavor the electrostatic PrP self-interaction. On the other hand, we observed that Ca^2+^ coordination sites were in fact surrounded by only weak positive MEP because the cationic +2 charge was shielded by the coordination of negatively charged residues. Based on this observation, we guessed if ligand exchange or reorganization of the coordination geometry may favor the interaction between two approaching Ca^2+^ coordination sites. According to the detected localization of Ca^2+^ coordination sites, this interaction mechanism would, again, allow the approach of region 1 and 3 in the self-aggregation of IV or V systems ([Ca^2+^] > 5 mM), even in the absence of electrostatic complementarity. Such a hypothesis was corroborated by quantum mechanical calculations evidencing how the condensation of reduced models of region 1 and 3 Ca^2+^ coordination sites is affected by a reaction free energy of only 1.5 kcal/mol (Fig 8, process (2)), consistent with a weakly left-shifted equilibrium. These calculations also showed that the free energy for the coordination a further carboxylate group at either DBM or another monocoordinated Ca^2+^ binding site is -26 or -21.5 kcal/mol, respectively (see Fig 8, process (1) and (3)) and, thus, suggest that [Ca^2+^] inducing the formation of DBM but leaving some uncoordinated sites on other regions, may probably be more effective in promoting the self-aggregation of PrP. Based on the outcomes of MD simulations, we stated that the Ca^2+^ binding residues Glu 200 and Glu 196 are not coordinated in IVb and Vb systems, respectively (S1 Table), thus leaving room to the Ca^2+^-mediated region 1-region 3 approach at either 10 or 20 mM.

To complete the analysis of the molecular properties controlling the self-aggregation properties of the considered PrP^C^ systems, the surrounding space of Ib, IIb, and IVb subsets was mapped by the use of DRY and HOH probes of GRID program [22] and the corresponding molecular interaction fields (MIFs) analyzed to locate the regions of space with higher hydrophobic and hydrophilic character, respectively. This procedure searches for MIF minima and, subsequently, identifies clusters of MIF minima that are spatially proximal, and allows to straightforwardly locate hydrophobic and hydrophilic regions in the space surrounding the PrP^C^ structure. Indeed, in the ideal self-aggregation of protein units, the hydrophobic clusters are expected to mutually overlap thus remaining buried in the aggregate structure, whereas hydrophilic clusters would favor the interunit interaction by either overlapping (protein-protein polar contacts) or being exposed to the bulk (protein-bulk polar contacts). Our analysis showed how E200K mutation not only induces electrostatic complementarity in region 1 and 3, but also favors a particularly effective hydrophobic/hydrophilic match of these regions. Indeed, we found that both the region 1 and 3 in IIb system present one hydrophobic and one hydrophilic clusters well exposed and at a similar reciprocal distance, i.e. in a spatial configuration that optimizes their overlapping (Fig 9). The alignment of E200K units according to the hydrophobic/hydrophilic overlapping of region 1 and 3 yielded “zig-zag” chains of PrP^C^ units which can then interact by the approach of other structural frames. In particular, we showed how the hydrophobic cluster on N-terminus derived from the disruption of its anchoring at H2 may approach to the hydrophobic cluster on H1 domain thus giving rise to a lateral interaction between E200K chains (Fig 9). This aggregation model for the E200K PrP^C^ was fully consistent with the region 1/region 3 electrostatic complementarity acquired by mutation and, in addition, allowed the mutual approach of all hydrophobic clusters. On the other hand, neither electrostatics nor MIF approach allowed to identify a similarly effective aggregation sketch for PrP^C^ wild type, thus remarking the importance of acquired or spontaneous (though very slow) structural alteration to promote self-aggregation. The Ca^2+^-bound PrP^C^ units presented, again, a different spatial configuration of MIF clusters compared to IIb units that did not allow their optimal overlapping. However, we observed that the Ca^2+^ coordination sites in region 1 and 3 are placed at the approximately same location of the overlapping hydrophobic and hydrophilic clusters found in the E200K aggregate. We also stated that either a free or Ca^2+^-bound carboxylate group may coordinate to a model of the DBM on region 1 in a strongly exoergonic or weakly endoergonic process, respectively, thus leaving room to a possible Ca^2+^-mediation in the PrP^C^ aggregation. By the alignment of Ca^2+^-bound PrP^C^ units obtained from IVb subset, we were able to show the feasibility of this coordination-mediated aggregation model based on two evidences: i) the Ca^2+^ coordination site on region 3, located on Glu 196, may superimpose with one of the metal centers of DBM (the one close to Asp 167) on region 1 thus giving rise to a coordination-mediated interaction (reaction (1), Fig 8) at a free energy cost of only 1.5 kcal/mol; ii) the carboxylate side chain on residue Glu 200 may approach and bind at the Ca^2+^ coordination site on Glu 219 (region 2), a process resembling reaction (3) of Fig 8 with a free energy gain of about -21.5 kcal/mol. As indicated by our calculations, the concomitance of reaction (1)-like and reaction (3)-like interfacing between region 1 and 3 is energetically favored in first approximation by 20 kcal/mol, and the hypothesis of a direct involvement of Ca^2+^ coordination in the mechanism of PrP^C^ aggregation for [Ca^2+^] > 5 mM seems to be thus corroborated. Again, we showed how both E200K mutation and Ca^2+^ binding trigger similar structural alterations such as either the local charge inversion on region 3 or the alteration/disruption of N-terminus anchoring on H2 domain, that lead to an aggregation-prone status of PrP^C^. On the other hand, electrostatics and molecular interaction fields analyses displayed how these two pathogenic stimuli seem to enhance the PrP^C^ aggregation propensity by different mechanisms: i) E200K units aggregate by region 1 and 3 electrostatic complementarity and optimal match of all hydrophobic regions; ii) Ca^2+^-bound units aggregate mainly by the match and rearrangement of Ca^2+^ coordination sites. As a final consideration, this computational work highlighted the importance of PrP model by evidencing how the extension of even a few residues, but leading to complete C-terminus and inclusion of a portion of the N-terminal neurotoxic peptide, may unveil structural effects otherwise undetectable.

## Conclusions

In this paper, we used computational approaches to elucidate the aggregation propensity of PrP^C^ protein systems in response to two pro-scrapie stimuli: [Ca^2+^] and E200K mutation. Two reduced segments of PrP, 125–228 (segment a) and 120–231 (segment b), were used to model the structure and the response to the selected stimuli of hPrP^C^(90–231) by a combination of molecular dynamics and molecular interaction fields analyses. The MD analysis of the more extended segment b led to unexpected evidences: i) the anchoring of N-terminus on H2 domain was detected in wild type model but resulted to be altered or disrupted when either E200K mutation or Ca^2+^ binding occurs; ii) a peculiar dinuclear Ca^2+^ binding motif (DBM) was detected in proximity of the C-terminus and involving the S2-H2 loop for [Ca^2+^] > 5 mM. The N-terminus detachment from the globular domain and the localization of positive charge in region 3 were identified as common structural features induced by both analyzed stimuli. The analysis of electrostatic and hydrophobic/hydrophilic character of the considered PrP^C^ systems allowed to pave hypotheses for their self-assembly. The E200K mutant showed both a marked electrostatic complementarity between region 1 (negative) and region 3 (positive) and the presence in these regions of one hydrophobic and one hydrophilic spot at approximately the same distance, leading to a particularly effective linear assembly via the region 1-region 3 interunit approach. The so-formed chains of E200K mutants can then interact by the match of hydrophobic spots placed on the N-terminus (formed by the alteration of its anchoring on H2) and on H1 domain. Our models showed that such a self-assembly scheme can not be routed by wild type units in which neither region 1-region 3 complementarity nor effective match of hydrophobic spots could be detected. On the other hand, Ca^2+^-bound units may undergo to a self-interaction process characterized by the interunit coordination of Ca^2+^ ion. In particular, we hypothesized the overlapping of a monocoordinated Ca^2+^ in region 3 with one of the two DBM metal centers in region 1 leading to a linear assembly scheme resembling that of E200K mutants. This coordination-mediated interaction of region 1 and 3 is further stabilized by the approach of either Glu 196 or Glu 200 carboxylate moiety to a Ca^2+^ coordination site on region 2. These results spotlight the structural frame formed by the S2-H2 loop and the C-terminus as a key determinant of the aggregation propensity of PrP^C^ and provide for a base of structural information, relying on electrostatics and hydrophobic/hydrophilic character of the PrP^C^ surface or its outer space, by which aggregation hypotheses can be drawn. On the other hand, neither electrostatics or hydrophobic effect are the only possible interactions driving the PrP^C^ self-assembly, as witnessed by the possible role of Ca^2+^ coordination in mediating the region 1-region 3 approach. Although the structural information and hypotheses on E200K- or [Ca^2+^]-induced PrP^C^ aggregation were obtained uniquely by computational outcomes, the present investigation may provide for new prompts of future experimental or computational works.

## Supporting Information

S1 Fig Trajectory stabilization. Root mean squared deviations of the backbone atom coordinates along the trajectory of (A) Ia-Va and (B) Ib-Vb systems. Root mean squared deviations of the charged residues atom coordinates along the trajectory of (C) Ia-Va and (D) Ib-Vb systems.
(TIF)

S2 Fig Anchoring of N-terminus at globule domain of PrP^C^. Reported systems: Ib (left), IIb (middle), and Vb (right).
(TIF)

S3 Fig Averaged MEP isosurfaces. From the top to below Ia, IIa, and Va (left) and Ib, IIb, and Vb (right). Positive and negative isosurfaces are depicted as blue and red meshes, respectively.
(TIF)

S4 Fig Octamer of wild type PrP^C^ units. Hydrophobic and hydrophilic clusters are depicted with yellow and red spheres, respectively. Also shown: interunit hydrophobic matches at the region 1-region 3 interface (purple dashed circles) and H1-H2 hydrophobic approach (dashed arrows).
(TIF)

S5 Fig Detailed view of the region 1-region 3 interface detected in the octamer of Ca^2+^-bound PrP^C^. (TIF)

S6 Fig Octamer of Ca^2+^-bound PrP^C^ units. Hydrophobic and hydrophilic clusters are depicted with yellow and red spheres, respectively. Also shown: interunit region 1-region 1 and H1-H2 hydrophobic approach (purple dashed circles); region1-region3 interface (green dashed circles).
(TIF)

S1 File Comments on S3 Fig. (DOCX)

S2 File Computational details. (DOCX)

S1 Table Percentage of trajectory with Ca^2+^-residue distance within 0.25 nm. (DOC)

S2 Table Clustering analysis (last 100 ns) based on the alignment and positional deviation of ionizable residues. (DOC)
