# Supplementary figures and images for: The Effects of Ca2+ Concentration and E200K Mutation on the Aggregation Propensity of PrPC: A Computational Study
Source: PLoS One. 2016 Dec 13;11(12):e0168039. doi: 10.1371/journal.pone.0168039 (PMC5154561; doi:10.1371/journal.pone.0168039)

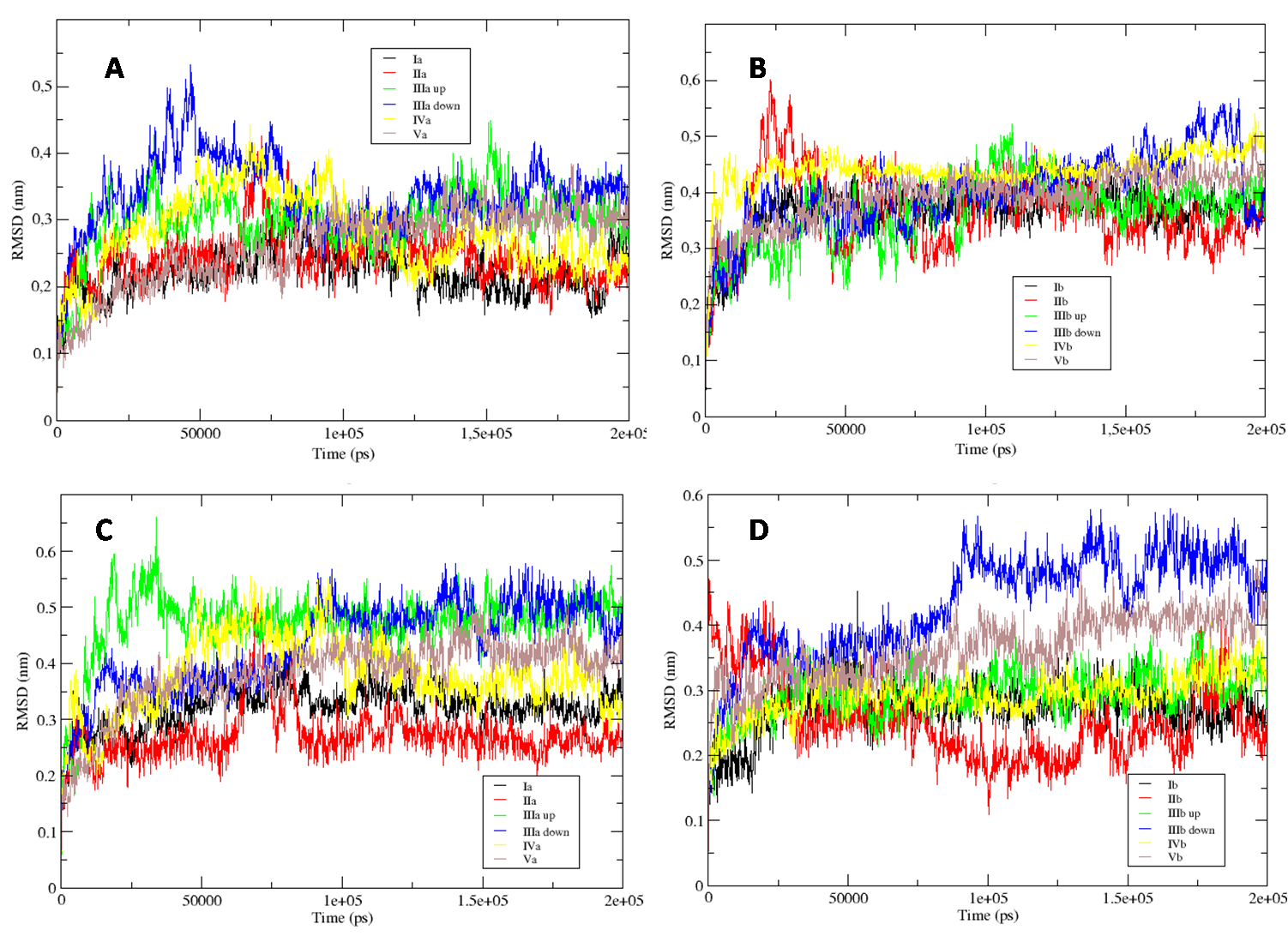

Supplement: S1 Fig — Root mean squared deviations of the backbone atom coordinates along the trajectory of (A) Ia-Va and (B) Ib-Vb systems. Root mean squared deviations of the charged residues atom coordinates along the trajectory of (C) Ia-Va and (D) Ib-Vb systems. (TIF) [file pone.0168039.s001.tif]

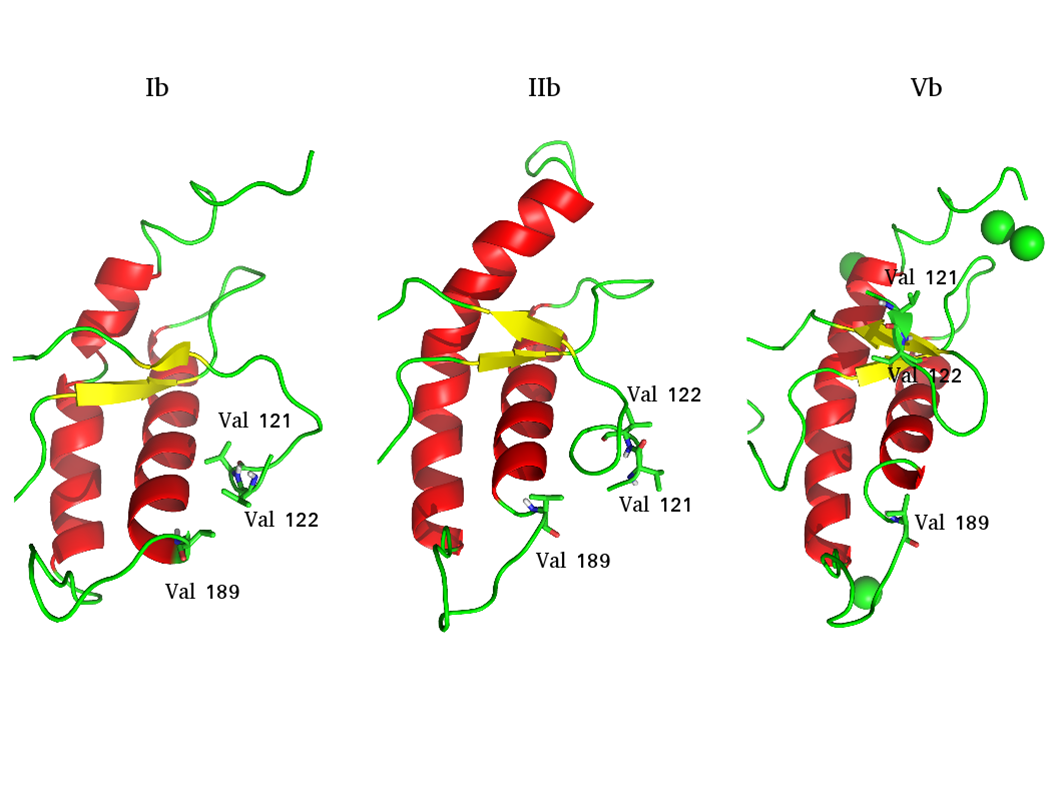

Supplement: S2 Fig — Reported systems: Ib (left), IIb (middle), and Vb (right). (TIF) [file pone.0168039.s002.tif]

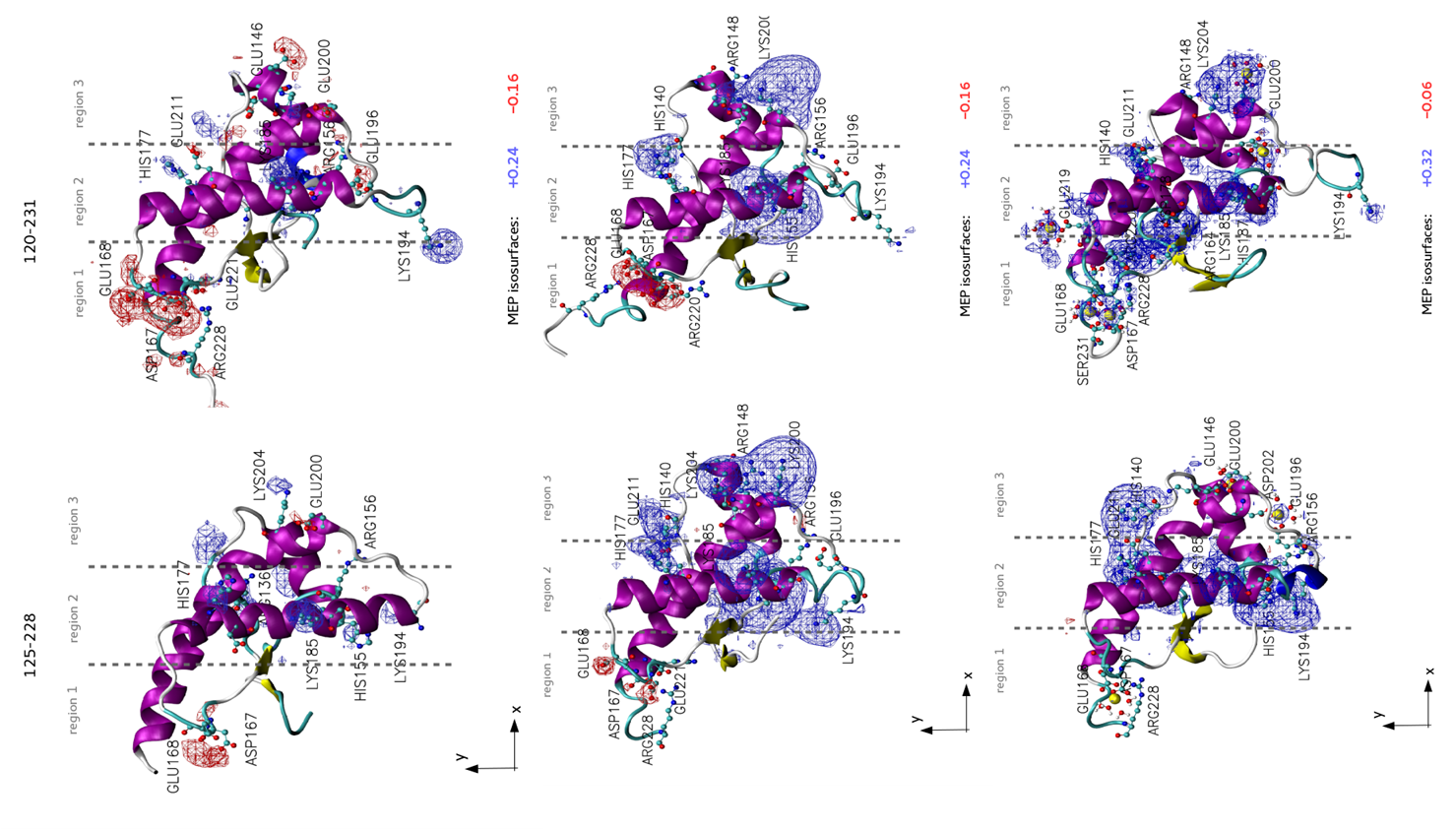

Supplement: S3 Fig — From the top to below Ia, IIa, and Va (left) and Ib, IIb, and Vb (right). Positive and negative isosurfaces are depicted as blue and red meshes, respectively. (TIF) [file pone.0168039.s003.tif]

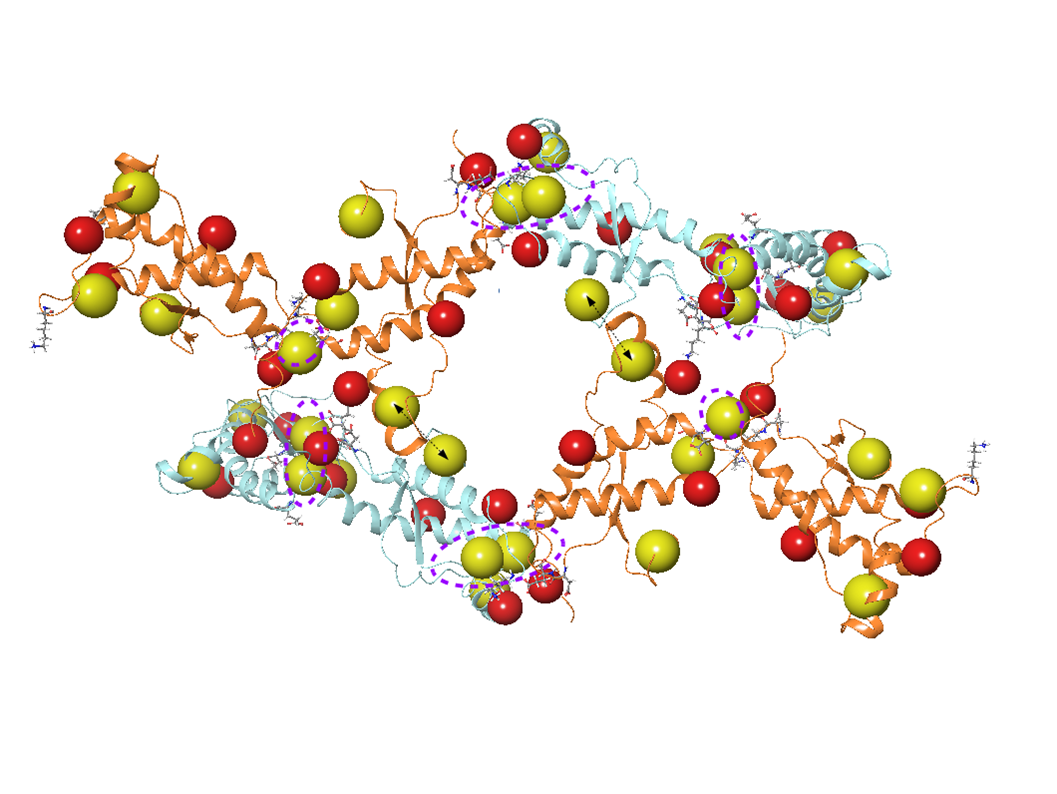

Supplement: S4 Fig — Hydrophobic and hydrophilic clusters are depicted with yellow and red spheres, respectively. Also shown: interunit hydrophobic matches at the region 1-region 3 interface (purple dashed circles) and H1-H2 hydrophobic approach (dashed arrows). (TIF) [file pone.0168039.s004.tif]

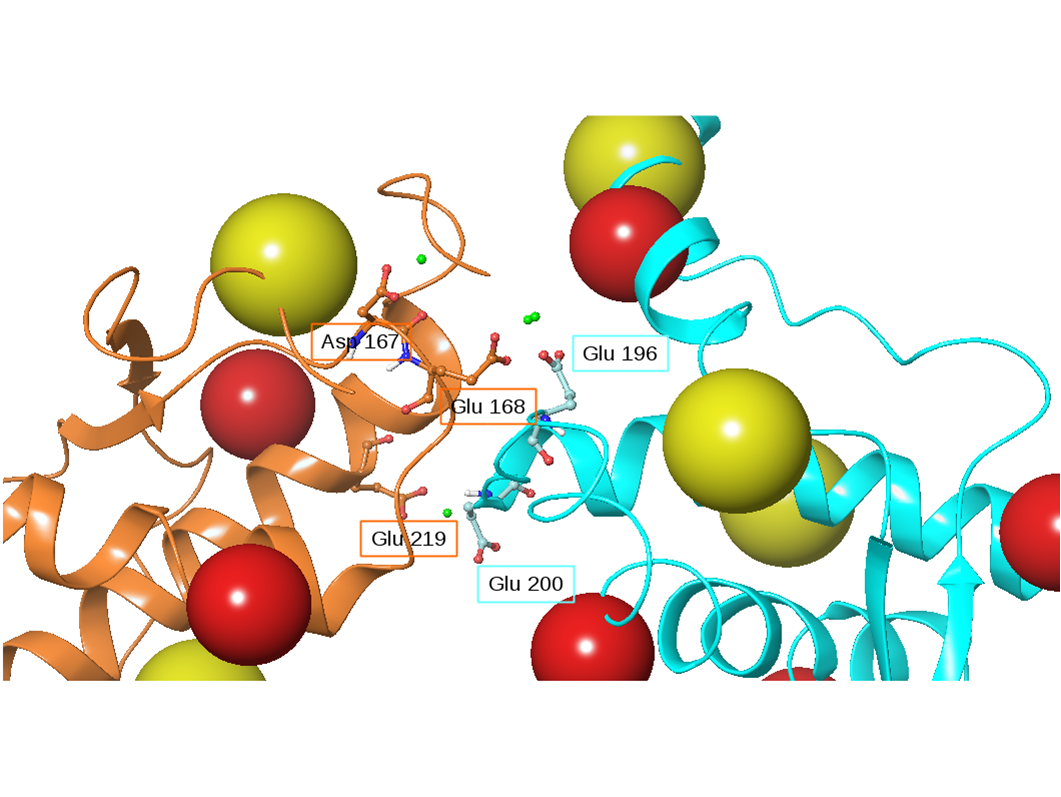

Supplement: S5 Fig — (TIF) [file pone.0168039.s005.tif]

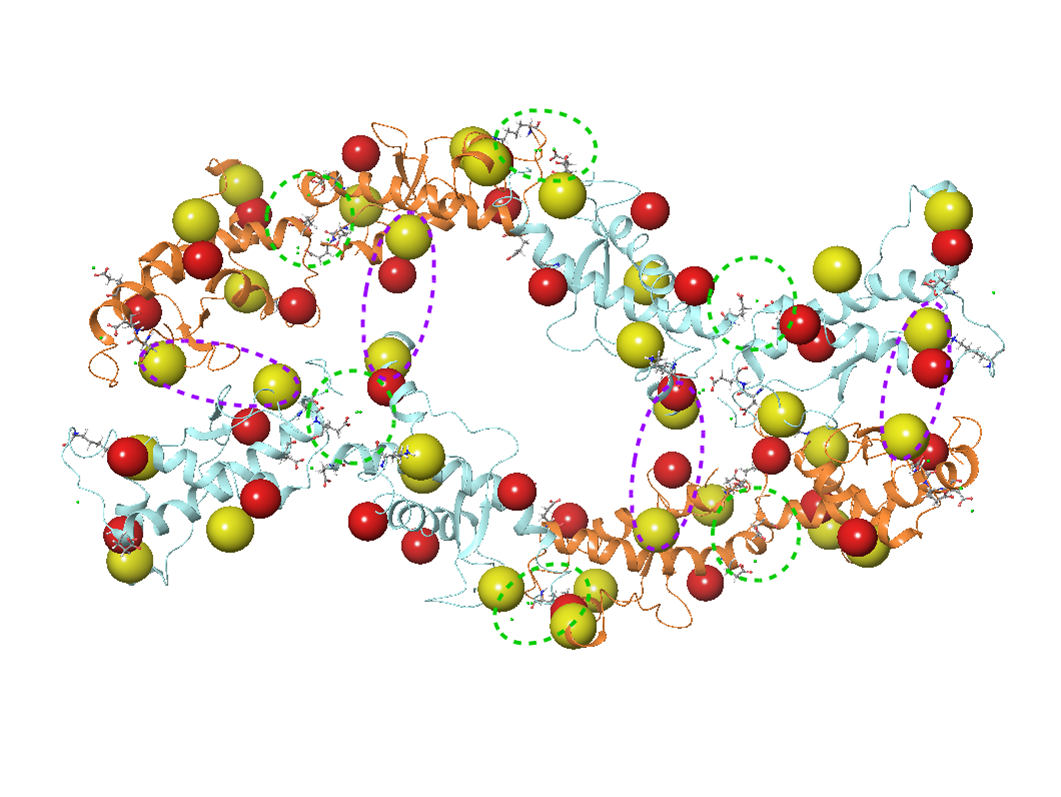

Supplement: S6 Fig — Hydrophobic and hydrophilic clusters are depicted with yellow and red spheres, respectively. Also shown: interunit region 1-region 1 and H1-H2 hydrophobic approach (purple dashed circles); region1-region3 interface (green dashed circles). (TIF) [file pone.0168039.s006.tif]
