# Supplementary material for: The Effects of Ca2+ Concentration and E200K Mutation on the Aggregation Propensity of PrPC: A Computational Study
Source: PLoS One. 2016 Dec 13;11(12):e0168039. doi: 10.1371/journal.pone.0168039 (PMC5154561; doi:10.1371/journal.pone.0168039)
Supplement: S1 File — (DOCX) [file pone.0168039.s007.docx]

**Comments on S3 Fig**

Wild type systems Ia and Ib displayed similarities in the distributions of surface charges as shown by the corresponding MEP isosurfaces. In particular, we evidenced a neat negatively charged site in region 1 originated by the rather exposed carboxyl side chains of vicinal Asp 167 and Glu 168 residues, as expected the negative charge density was higher in Ib by the contribution of the C-terminus carboxyl group of Ser 231. Region 2 disclosed a positive charge character in both systems, whereas more appreciable differences were detected in region 3. While negative and positive charges were seemingly balanced in Ia, a slightly more negative character of region 3 was detected in Ib system, mainly originated from Glu 146 and Glu 200 side chains.
