# Supplementary material for: The Effects of Ca2+ Concentration and E200K Mutation on the Aggregation Propensity of PrPC: A Computational Study
Source: PLoS One. 2016 Dec 13;11(12):e0168039. doi: 10.1371/journal.pone.0168039 (PMC5154561; doi:10.1371/journal.pone.0168039)
Supplement: S2 File — (DOCX) [file pone.0168039.s008.docx]

**Computational details**

**Molecular dynamics simulations and analyses**

The experimental NMR structures of recombinant wild type and E200K mutant of PrP available from the PDB archives (1QM0 and 1FKC) [Manuscript 14, 1] were used as starting models of the 125-228 segment in molecular dynamics (MD) simulations performed with the Gromacs package [2]. The NMR structure coded 2lsb [3] was used to generate a starting model for the 120-231 segment of the wild type PrP^C^, whereas the corresponding E200K mutated segment was obtained from the wild type model after the conversion of residue 200 from GLU to LYS performed in the Maestro workspace [4]. The starting models of PrP set for the subsequent MD simulations were reported and labelled (See Table 1). In each case, the protein molecule was placed in a cubic box whose dimension prevents self-interaction, and solvated with up to 13236-13246 water molecules (TIP3 [5]) at the typical density of liquid water at 298 K and 1.0 atm. For systems Ia and Ib, electrical neutrality was obtained by adding Na^+^ and Cl^–^ counterions. Instead, Ca^2+^ and Cl^–^ counterions were added to gain electrical neutrality and to simulate the incubation of wild type systems in aqueous solutions at [CaCl_2_] of 5 (IIIa and IIIb), 10 (IVa and IVb), and 20 (Va and Vb). For both IIIa and IIIb, electroneutrality required only one Ca^2+^ ion. To better assay the Ca^2+^ binding properties of PrP, two replica of both IIIa and IIIb systems were generated by the placement of Ca^2+^ into two different regions of the PrP surrounding space: i) in IIIa1 and IIIb1 systems, Ca^2+^ was randomly placed in the space formed by region 1 plus one half (proximal to region ) of region 2 ; ii) in IIIa2 and IIIb2 systems, Ca^2+^ was randomly placed in the space formed by region3 plus the other half of region 2 (See Results, Fig 4). Therefore, because the total charges of 125-228 and 120-231 segments of PrP are -1 and -2, respectively, we eventually obtained: i) IVa and IVb systems with 3 and 4 Ca^2+^ ions, respectively; ii) IVa and IVb systems with 5 and 6 Ca^2+^ ions, respectively. All simulations were performed adopting the same computational scheme: i) local energy minimization; ii) slow heating up: six MD runs (500 ps) in which the protein temperature was set at 0, 100, 150, 200, 250, and 300 K; ii) production runs of 200 ns at 300 K in an isothermal/isobaric ensemble, using the velocity rescaling scheme (temperature) and the isotropic Berendsen coupling scheme (pressure) [6]. All protein systems were simulated in the OPLS-2005 force field [7, 8], the LINCS algorithm was adopted to constrain all bond lengths [9], and the long range electrostatics were computed by the Particle Mesh Ewald method [10]. Trajectory analyses were carried out by using suitable Gromacs utilities with the support of either VMD or Maestro graphical interfaces [4, 11]. Ensembles of 1000 protein conformations per trajectory (one per 100 ps) were extracted from the last 100 ns and clusterized by using the *g_cluster* utility. The clustering method labelled *gromos* [12] was employed to sample a subset of representative configurations of the system based on the diverse distribution of charged protein residues. At this purpose, all protein conformations were superimposed by minimizing the root mean square distances between the atoms of ASP, GLU, ARG, LYS and HIS residues. A clustering cut-off of 0.15 nm was employed in the analysis of all ensembles. The middle structures of the most populated clusters, i.e. at least 80% coverage of the whole ensemble, were used to generate the representative subset of each system. Then, each subset element was optimized in the OPLS-2005 force field [7, 8] and by the use of the GB/SA polarisable continuum method [13, 14] for the simulation of the aqueous medium with the MacroModel software [15]. All the water molecules and ions of the bulk were removed with the exception of Ca^2+^ within 3.0 Å of any protein atom together with the water molecules in the first coordination sphere. To prevent from a substantial loss of MD structural information, the Cartesian coordinates of alpha-carbon atoms were frozen and loose convergence criteria were applied (maximum 10000 of LBGFS iterations, 0.15 kJ mol^–1^ Å^–1^ gradient threshold). All subsets were aligned to the same space of coordinate by the minimization of the spatial root mean squared deviations of ionizable residues (ASP, GLU, ARG, LYS and HIS) using the first element (i.e. the most representative) of Ia subset as reference. After this alignment procedure, all protein systems resulted to share the same principal axis, i.e. x axis, approximately collinear with the H2 domain major axis.

**Molecular interaction fields calculation and analysis**

The GRID program was used to calculate the molecular interaction field (MIF) of either DRY and HOH probe for each protein configuration [Manuscript 22, Manuscript 23]. The weights assigned to each subset element were used to calculate a final weighted average DRY-MIF or HOH-MIF which maps the hydrophobicity or hydrophilic, respectively, in the surrounding space of the whole subset. All MIF computations were performed using a parallel version of the code based on POSIX thread [Manuscript 16] previously tested and briefly discussed [Manuscript 18]. The molecular electrostatic potential (MEP) was mapped in the same spatial grids to calculate the Carbò similarity between the various multiconformational models by using the comparison schemes reported elsewhere [Manuscript 11, Manuscript 18]. MEP similarity profiles were calculated for all possible pairs of conformations within two subsets and, then, averaged to obtain an overall similarity profile. Each point of the final profile is therefore the weighted average of Carbò index values, we can also assigned a standard deviation value that yields an indication of the MEP similarity “stability” in a region of space. The average MEP of each protein system was also calculated by using the weights assigned to each subset element. Finally, both the MEP similarity profile calculations and the DRY MIF comparisons were performed using specifically designed in-house programs developed in Python [Manuscript 20]. The implemented Python procedures make use of mainly two modules: NumPy [17] and Pybel [18].

**DFT calculations**

All calculations were performed by using the Gaussian 09 programs package [19]. The local minimum geometry of each species (see below) was calculated with the hybrid exchange-correlation functional B3LYP [20] - expected to be particularly suitable for the systems in object [21] - and the following scheme of all-electrons Pople basis sets [22]: i) the double-zeta plus d polarization and s diffusion (6-31+G*) for the treatment of Ca atom; ii) the double-zeta plus d or p polarization (6-31G(d,p))for the treatment of C, N, O or H atom, respectively. The conductor-like screening model implemented in Gaussian 09 (CPCM [23]) was used in the calculation of local minima to simulate the effect of a water bulk (default setting for water, See Gaussian 09 technical notes). Vibrational frequencies analyses were performed at the same level of theory (CPCM/B3LYP/6-31G*) to confirm the correct nature of the optimized stationary points and calculate zero-point energy and thermal corrections (under the hypothesis of an ideal gas behavior) for enthalpy and free energy estimations.

**Computational detail’s references**

1. Zahn R, Liu A, Luhrs T, Riek R, von Schroetter C, Garcia FL, Billeter M, Calzolai L, Wider G, Wüthrich K NMR solution structure of the human prion protein. Proc. Natl. Acad. Sci. USA 2000; 97: 145–150.
2. Van Der Spoel D, Lindahl E, Hess B, Groenhof G, Mark AE, Berendsen HJ Fast Parallel Algorithms for Short-Range Molecular Dynamics. J. Comput. Chem., 2005; 26: 1701-1718.
3. Biljan I et al. Structural basis for the protective effect of the human prion protein carrying the dominant-negative E219K polymorphism. Biochem. J. 2012; 446: 243-251.
4. Schrödinger LLC (2013) Maestro, Version 9.4. New York, NY, USA.
5. Berendsen HJC, Postma JPM, van Gunsteren WF, Hermans J in Intermolecular forces; B. Pullmann, Ed.; Reider Publishing Company: Dordrecht, 1981; pp. 331–342.
6. Berendsen HJC, Postma JPM, van Gunsteren WF, Di Nola A, Haak JR Molecular dynamics with coupling to an external bath. J. Chem. Phys. 1984; 81: 3684-3690.
7. Jorgensen WL, Maxwell DS, Tirado-Rives J Development and testing of the OPLS all-atom force field on conformational energetics and properties of organic liquids. J. Am. Chem. Soc. 1996; 118: 11225-11236.
8. Kaminski GA, Friesner RA, Tirado-Rives J, Jorgensen WJ Evaluation and Reparametrization of the OPLS-AA Force Field for Proteins via Comparison with Accurate Quantum Chemical Calculations on Peptides. J. Phys. Chem. B 2001; 105: 6474-6487.
9. Hess B, Bekker H, Berendsen HJC, Frajie JCEM LINCS: a linear constraint solver for molecular simulations. J. Comput. Chem. 1997; 18: 1463-1472.
10. Darden TA, York DM, Pedersen LG Particle mesh Ewald: An N⋅ log (N) method for Ewald sums in large systems. J. Chem. Phys. 1993; 98: 10089-10092.
11. Humphrey W, Dalke A, Schulten K VMD: visual molecular dynamics. J. Molec. Graphics 1996; 14: 33-38.
12. Daura X, Gademann K, Jaun B, Seebach D, van Gunsteren WF, Mark AE Peptide folding: when simulation meets experiment. Angew. Chem. Int. Ed. 1999; 38: 236–240.
13. Still WC, Tempczyk A, Hawley RC, Hendrickson T Semianalytical treatment of solvation for molecular mechanics and dynamics. J. Am. Chem. Soc. 1990; 112: 6127-6129.
14. Di Qiu M, Shenkin P, Hollinger F, Still W The GB/SA continuum model for solvation. A fast analytical method for the calculation of approximate Born radii. J. Phys. Chem. A 1997; 101: 3005–3014.
15. Schrödinger, LLC (2010), MacroModel, version 9.8, New York, NY.
16. Butenhof DR, Programming with POSIX threads, Addison-Wesley Longman Publishing Co., Inc. Boston, MA, USA (1997).
17. Jones E, Oliphant E, Peterson P, et al. SciPy: Open Source Scientific Tools for Python, 2001-,<http://www.scipy.org/>, Online; accessed 2015-02-17.
18. O'Boyle NM, Morley C, Hutchison GR Pybel: a Python wrapper for the OpenBabel cheminformatics toolkit. Chem. Cent. J., 2008; 2: 5.
19. Gaussian 09, Revision A.02, M. J. Frisch, G. W. Trucks, H. B. Schlegel, G. E. Scuseria, M. A. Robb, J. R. Cheeseman, G. Scalmani, V. Barone, B. Mennucci, G. A. Petersson, H. Nakatsuji, M. Caricato, X. Li, H. P. Hratchian, A. F. Izmaylov, J. Bloino, G. Zheng, J. L. Sonnenberg, M. Hada, M. Ehara, K. Toyota, R. Fukuda, J. Hasegawa, M. Ishida, T. Nakajima, Y. Honda, O. Kitao, H. Nakai, T. Vreven, J. A. Montgomery, Jr., J. E. Peralta, F. Ogliaro, M. Bearpark, J. J. Heyd, E. Brothers, K. N. Kudin, V. N. Staroverov, R. Kobayashi, J. Normand, K. Raghavachari, A. Rendell, J. C. Burant, S. S. Iyengar, J. Tomasi, M. Cossi, N. Rega, J. M. Millam, M. Klene, J. E. Knox, J. B. Cross, V. Bakken, C. Adamo, J. Jaramillo, R. Gomperts, R. E. Stratmann, O. Yazyev, A. J. Austin, R. Cammi, C. Pomelli, J. W. Ochterski, R. L. Martin, K. Morokuma, V. G. Zakrzewski, G. A. Voth, P. Salvador, J. J. Dannenberg, S. Dapprich, A. D. Daniels, O. Farkas, J. B. Foresman, J. V. Ortiz, J. Cioslowski, and D. J. Fox, Gaussian, Inc., Wallingford CT, 2009.
20. Becke AD, “Density-functional thermochemistry. III. The role of exact exchange,” J. Chem. Phys., 98 (1993) 5648-52.
21. Niu SQ, Hall MB Theoretical studies of reactions of transition-metal complexes. Chem .Rev. 2000; 100: 353–405.
22. Ditchfield R, Hehre WJ, Pople JA, Self-Consistent Molecular Orbital Methods. 9. Extended Gaussian-type basis for molecular-orbital studies of organic molecules J. Chem. Phys. 1971; 54: 724. Francl MM, Pietro WJ, Hehre WJ, Binkley JS, DeFrees DJ, Pople JA, Gordon MS, Self-Consistent Molecular Orbital Methods. 23. A polarization-type basis set for 2nd-row elements J. Chem. Phys. 1982; 77: 3654-65. Rassolov VA, Ratner MA, Pople JA, Redfern PC, Curtiss LA, 6-31G* Basis Set for Third-Row Atoms J. Comp. Chem. 2001; 22: 976-84.
23. Cossi M, Rega N, Scalmani G, Barone V, Energies, structures, and electronic properties of molecules in solution with the C-PCM solvation model J. Comp. Chem. 2003; 24: 669-81.
