# Supplementary material for: The Effects of Ca2+ Concentration and E200K Mutation on the Aggregation Propensity of PrPC: A Computational Study
Source: PLoS One. 2016 Dec 13;11(12):e0168039. doi: 10.1371/journal.pone.0168039 (PMC5154561; doi:10.1371/journal.pone.0168039)
Supplement: S1 Table — (DOC) [file pone.0168039.s009.doc]

**S1 Table. Percentage of trajectory with Ca^2+^-residue distance within 0.25 nm.**

| residue | IIIa1 | IIIb1 | IIIa2 | IIIb2 | IVa | IVb | Va | Vb |
| --- | --- | --- | --- | --- | --- | --- | --- | --- |
| Glu 146 | 0 | 0 | 0 | 0 | 80 | 0 | 0 | 0 |
| Glu 152 | 0 | 0 | 95 | 0 | 0 | 0 | 0 | 90 |
| Asp 167 | 99 | 100 | 0 | 0 | 98 | 100 | 70 | 96 |
| Glu 168 | 92 | 99 | 0 | 0 | 95 | 100 | 88 | 100 |
| Glu 196 | 0 | 0 | 0 | 0 | 0 | 68 | 95 | 0 |
| Glu 200 | 0 | 0 | 0 | 97 | 0 | 0 | 85 | 98 |
| Glu 207 | 0 | 0 | 0 | 0 | 0 | 0 | 30 | 0 |
| Glu 219 | 0 | 0 | 0 | 0 | 90 | 82 | 0 | 68 |
| Glu 221 | 0 | 0 | 0 | 0 | 0 | 0 | 98 | 0 |
| Ser 231 | 0 | 0 | 0 | 0 | 0 | 100 | 0 | 99 |
