# Supplementary material for: The Effects of Ca2+ Concentration and E200K Mutation on the Aggregation Propensity of PrPC: A Computational Study
Source: PLoS One. 2016 Dec 13;11(12):e0168039. doi: 10.1371/journal.pone.0168039 (PMC5154561; doi:10.1371/journal.pone.0168039)
Supplement: S2 Table — (DOC) [file pone.0168039.s010.doc]

**S2 Table. Clustering analysis (last 100 ns) based on the alignment and positional deviation of ionizable residues.**

| systems | average RMSD | #clusters | ?#clusters | #representative |
| --- | --- | --- | --- | --- |
| Ia \ Ib | 0.2163 \ 0.1697 | 54 \ 18 | 0 \ 0 | 11 \ 2 |
| IIa \ IIb | 0.2072 \ 0.2282 | 37 \ 70 | −14 \ +52 | 7 \ 19 |
| IIIa \ IIIb up | 0.2250 \ 0.1901 | 119 \ 30 | +65 \ +12 | 35 \ 5 |
| IIIa \ IIIb down | 0.2635 \ 0.2502 | 70 \ 60 | +16 \ +42 | 16 \ 13 |
| IVa \ IVb | 0.2287 \ 0.1928 | 83 \ 35 | +29 \ +17 | 25 \ 7 |
| Va \ Vb | 0.2252 \ 0.2381 | 86 \ 34 | +32 \ +16 | 24 \ 6 |
